# Tranexamic Acid Inhibits Angiogenesis and Melanogenesis *in Vitro* by Targeting VEGF Receptors

**DOI:** 10.7150/ijms.44188

**Published:** 2020-03-25

**Authors:** Jian-Wei Zhu, Ya-Jie Ni, Xiao-Yun Tong, Xia Guo, Xiao-Ping Wu, Zhong-Fa Lu

**Affiliations:** 1 Department of Dermatology, Zhejiang Hospital, No. 12, Lingyin Rd., Hangzhou, 310013, Zhejiang Province, China.; 2 Department of Dermatology, Second Affiliated Hospital, Zhejiang University School of Medicine, No. 88, Jiefang Rd., Hangzhou, 310009, Zhejiang Province, China.

**Keywords:** tranexamic acid, VEGF receptors, human umbilical vein endothelial cells, angiogenesis, melanocytes, melanogenesis

## Abstract

Melasma is a common but complex skin condition concerning cosmetic problems. Tranexamic acid (TA) has been proved to be effective in treatment of melasma with still unclear mechanisms. Here, we show that VEGF165 enhanced the expression of VEGF receptors (VEGFRs, including VEGFR-1, VEGFR-2 and NRP-1) in human umbilical vein endothelial cells (HUVECs), which was attenuated by TA. VEGF165 also promoted tyrosine phosphorylation of VEGFR-1 and VEGFR-2 in HUVECs, which was again abolished by TA. TA further showed similar effects to neutralization of VEGFR-1 and VEGFR-2 in inhibiting cell proliferation, migration, invasion and tube formation of HUVECs induced by VEGF165, suggesting that TA could inhibit angiogenesis by targeting VEGFRs in HUVECs. In addition, VEGF165 enhanced the expression of VEGFRs and promoted tyrosine phosphorylation of VEGFR-1 and VEGFR-2 in normal human melanocytes, which were also attenuated by TA. Furthermore, TA showed similar effects to neutralization of VEGFR-1 and VEGFR-2 in inhibiting tyrosinase activity, melanin production and even melanogenic proteins induced by VEGF165, suggesting that TA could reduce melanogenesis via inhibiting activation of VEGFRs and subsequent expression of melanogenic proteins in melanocytes. Taken together, we demonstrate that TA can inhibit angiogenesis and melanogenesis *in vitro* at least in part by targeting VEGFRs, which may offer a new understanding of the pathogenesis of melasma as well as the molecular mechanism for TA in treatment of the disease.

## Introduction

Melasma, formerly known as chloasma, is a common acquired dermatological condition characterized with hyperpigmentation and increased dermal capillaries in the lesional skin, typically occurring symmetrically on the face, with higher prevalence in females and darker skin types 1. Although common, the management of this disorder remains challenging given the incomplete understanding of the pathogenesis, its chronicity and recurrence rates 2. In addition to traditional treatments for melasma, there are also promising new treatments, including topical, oral and procedural therapies, among which oral tranexamic acid (TA) is now proved to be effective for melasma and ultraviolet (UV)-induced hyperpigmentation 2, 3. Localized microinjection of TA also improved melasma *in vivo* 4. However, it still remains unclear how TA exactly works. TA can prevent the binding of plasminogen originating from endothelial cells to keratinocytes, which is thought to be a possible mechanism for melasma treatment 5, implicating those interactions between the altered cutaneous vasculature and the overlying epidermis may have an influence on the development of hyperpigmentation of melasma.

Keratinocytes are the main epidermal cells in the skin. Vascular endothelial growth factor (VEGF) constitutes an important keratinocyte-derived factor in response to UV irradiation and acts in both autocrine and paracrine manner affecting epidermal cells and dermal tissues through binding with VEGF receptors (VEGFRs) 6-9. Epidermal keratinocytes had been demonstrated to express VEGFRs and co-receptors, and VEGF/VEGFR-2 autocrine signaling had been detected in keratinocytes 10, 11, even in epidermal appendages 12-16. UV could activate VEGFRs in normal keratinocytes, and the activated VEGFR-1 and VEGFR-2 signaling was involved in the pro-survival mechanism 17. Moreover, the over- expressed VEGFRs in psoriatic epidermis could be attenuated by narrow-band UVB therapy 18. Thus, VEGFRs are believed to participate in physiological and pathological conditions in the epidermis.

Although keratinocytes account for the main epidermal cells in the skin, melanocytes, situated at the basal layer of the epidermis, are responsible for melanin synthesis and skin color. One of the most specific and potent environmental factors associated with skin color is UVB 8, which induces pigmentation by controlling several parameters of melanocyte differentiation such as melanin synthesis, dendrite outgrowth, and melanosome transport required for melanin distribution within the epidermis 19. Melanogenesis is a common cellular process, whereas hyperfunction of melanocytes can also result in pigmentary disorders such as melasma 20. Melanocytes in the lesional melasma epidermis are generally enlarged, have prominent dendrites and increased melanosomes, resulting in a major clinical feature as hyperpigmented patches, but patients have additional distinguishing features like pronounced telangiectatic erythema basically confined to the melasma lesional skin 21. These features were confirmed by significant increase in vascularity in the dermis and VEGF over-expression in the epidermis in melasma lesions compared with those in perilesional normal skin 22, suggesting that a connection between vessels and cutaneous pigmentation could exist. Human melanocytes may respond to angiogenic factors because normal human melanocytes express functional VEGFRs, and the expression of VEGFR-2 was up-regulated by UVB 23. However, the influence of VEGFRs on physiological function of melanocytes especially melanogenesis needs further clarification.

Therefore, in this report, we investigated the functional significance of VEGFRs in human umbilical vein endothelial cells (HUVECs) and epidermal melanocytes in response to TA. Our results demonstrate that VEGFRs signaling is involved in angiogenesis and melanogenesis, and TA potently suppresses both angiogenic and melanogenic processes via inhibiting activation of VEGFRs respectively in HUVECs and normal human melanocytes, which may offer a new therapeutic target for hyperpigmented diseases like melasma.

## Materials and Methods

### Cell culture

HUVECs are purchased from the China Infrastructure of Cell Line Resources (Beijing, China) and were cultured in endothelial basal medium containing 1% fetal bovine serum (FBS) and endothelial cell growth medium 2 Bullet Kit in a humidified atmosphere of 5% CO_2_ at 37°C. Passages 3 and 5 were used in all experiments.

For melanocytes, adolescent foreskin was obtained from urinary surgery and handled aseptically. After removing subcutaneous elements, the specimen was cut into 0.5 cm^2^ pieces and put into 0.5% dispase (Gibco, Invitrogen, Auckland, USA) for overnight incubation at 4°C. The epidermis was peeled off from the dermis. The epidermis was further incubated in 0.25% trypsin for 10 min at 37°C. Trypsin activity was neutralized by adding FBS. The mixed cell suspension was filtered through nylon gauze (100 μm mesh) and cells were washed twice with 0.1 M phosphate-buffered saline (PBS) prior to resuspension in M2 medium (PromoCell, Heidelberg, Germany) supplemented with penicillin (100 units/ml) and streptomycin (100 mg/ml). Cells were seeded onto 10 cm culture dishes (Corning, Corning NY, USA), maintained at 37°C in a humidified atmosphere containing 5% CO_2_. Passages 3 to 5 were used in all experiments, including cell identification by Dopa staining and S-100 protein immunohistochemical staining.

### Western blot analysis

Cells were treated with VEGF165 and TA when grown to 80% confluent. Total cellular protein was extracted with lysis buffer (Beyotime Biotechnology, Beijing, China). An aliquot of proteins were separated by 10% sodium dodecyl sulfate-polyacrylamide gel and transferred onto the polyvinylidene difluoride membranes (Millipore, Bedford, MA). After blocking for 30 min, the membrane was incubated with primary antibodies for 2 h at room temperature, and then conjugated with horseradish peroxidase (HRP)-conjugated secondary antibodies (Jackson Laboratories, West Grove, PA, USA) (1:5000 dilution) for 1 h. Immunoreactivity was detected by ECL plus luminal solution (Amersham Biosciences, Piscataway, NJ, USA). Immunoreactive bands were detected using an enhanced chemiluminescence system (Millipore). Protein density was measured by a Bio-Rad XRS chemiluminescence detection system (Bio-Rad, USA). The blots shown are representative of at least three repeats. The primary antibodies used in this study were antibodies against VEGFR-1, VEGFR2, NRP-1 (1:500-1:1000 dilution) (Santa Cruz, CA, USA), phospho-VEGFR-1 (Y1213), phospho-VEGFR-2 (Tyr1175) (1:500 dilution) (Cell Signaling Technology, Danvers, MA, USA), MITF, Tyrosinase, Trp-1, Trp-2 (1:1000 dilution) and β-actin (1:5000 dilution) (Abcam, Shanghai, China).

### Cell proliferation assay

Cell proliferation was measured by MTT assay. Briefly, cells were plated in collagen-coated 96-well plates (5×10^4^ cells/ml, 100 μl/well) and cultured overnight. Cells were then stimulated with 0 or 10 ng/ml VEGF165 for 48 h, with or without incubation of 1 mg/ml TA, 5 μg/ml VEGFR-1 and/or VEGFR-2 neutralizing antibody. 20 μl of 3-[4,5-dimethylthiazol-2-yl]2,5-diphenyltetrazolium bromide (MTT; Sigma, St. Louis, MO, USA) at a final concentration of 5 mg/ml in PBS was incubated with the analyzed cells for 4 h at 37°C. Dark blue formazan crystals that formed in the living cells were dissolved by adding 100 μl of dimethylsulfoxide. The optical absorbency was measured at 570 nm as A value with a spectrophotometric reader Elx808 (Bio-Tek, Winooski, VT, USA).

### Wound Healing Assay

HUVECs were seeded in 6-well plates until confluent. A mechanical wound was created by gently scratching the cells with the tip of a pipette (time 0 hour). The cells were then washed with serum-free medium and cultured in endothelial cell growth medium containing 0.1% FBS with or without incubation of 10 ng/ml VEGF165, 1 mg/ml TA, 5 μg/ml VEGFR-1 and/or VEGFR-2 neutralizing antibody. Images were captured after 48 hours, and the relative migration distance was calculated using the following formula: the relative migration distance (%) =100 (AX-BX)/(A blank-B blank), where A is the width of the cell wound before incubation and B is the width of the cell wound after incubation.

### Transwell Invasion Assay

The assay was performed using chambers containing polycarbonate filters with pore size 8 μm (Merck Millipore, Darmstadt, Germany). The upper side of a polycarbonate filter was coated with Matrigel (BD Biosciences, Franklin Lakes, New Jersey) to form a continuous, thin layer. HUVECs (1× 10^5^) were resuspended in 300 μl of 0.1% FBS medium and then added to the upper chamber. The lower chamber was filled with 200 μl endothelial cell growth medium containing 0.1% FBS, with or without incubation of 10 ng/ml VEGF165, 1 mg/ml TA, 5 μg/ml VEGFR-1 and/or VEGFR-2 neutralizing antibody. After a 48-h incubation and removal of the cells from the upper chamber of the filter with a cotton swab, the cells on the underside were stained with 1% crystal violet and then counted under microscope.

### Tube Formation Assay

Plates with 24 wells were first coated with Matrigel (BD Biosciences). Unpolymerized Matrigel was placed in the wells (300 μl/well) and allowed to polymerize for 1 h at room temperature. HUVECs in 500 μl medium were seeded onto the polymerized Matrigel at a density of 5 ×10^4^ cells/well. After incubation with or without 10 ng/ml VEGF165, 1 mg/ml TA, 5 μg/ml VEGFR-1 and/or VEGFR-2 neutralizing antibody for 48 h, images of tube formation were acquired with an inverted phase- contrast light microscope (Olympus Corporation, Tokyo, Japan). The degree of tube formation was quantified in 5 random fields from each well using ImageJ software.

### Tyrosinase activity

Tyrosinase enzyme activity was estimated by measuring the rate of L-DOPA oxidation. Briefly, melanocytes were treated with 10 ng/ml VEGF165 for 48 h with or without incubation of 1 mg/ml TA, 5 μg/ml VEGFR-1 and/or VEGFR-2 neutralizing antibody. The cells were solubilized with phosphate buffer (pH 6.8) containing 1% Triton X-100. The cells were then sonicated, and the lysates were clarified by centrifugation at 18,000 × g for 15 min. After protein quantification and adjustment of protein concentrations with lysis buffer, 170 μL of 2 mM L-DOPA were added to each 96-well plate. The absorbance was measured spectrophotometrically at 470 nm following a 15-min incubation period at 37 °C. Tyrosinase activity was expressed as a fold change of the treated group vs. the control group.

### Melanin content assay

To determine the melanin content, melanocytes were treated with 10 ng/ml VEGF165 for 48 h with or without incubation of 1 mg/ml TA, 5 μg/ml VEGFR-1 and/or VEGFR-2 neutralizing antibody, after which they were washed with PBS and harvested. After centrifugation at 18,000 × g for 5 min, the harvested cell pellets were solubilized in boiled 1 N NaOH (65 °C) for 2 h, and color was analyzed at 405 nm using a microplate reader. Melanin content was expressed as a fold change of the treated group vs. the control group.

### Statistical analysis

Results are expressed as means ± SD. All experiments were performed at least in triplicate. Statistical analysis was performed using one-way analysis of variance (ANOVA) and Student's t-test. The differences were considered statistically significant when p value was less than 0.05.

## Results

### TA inhibited cell growth of HUVECs and melanocytes without cellular toxicity

**Figure 1:**
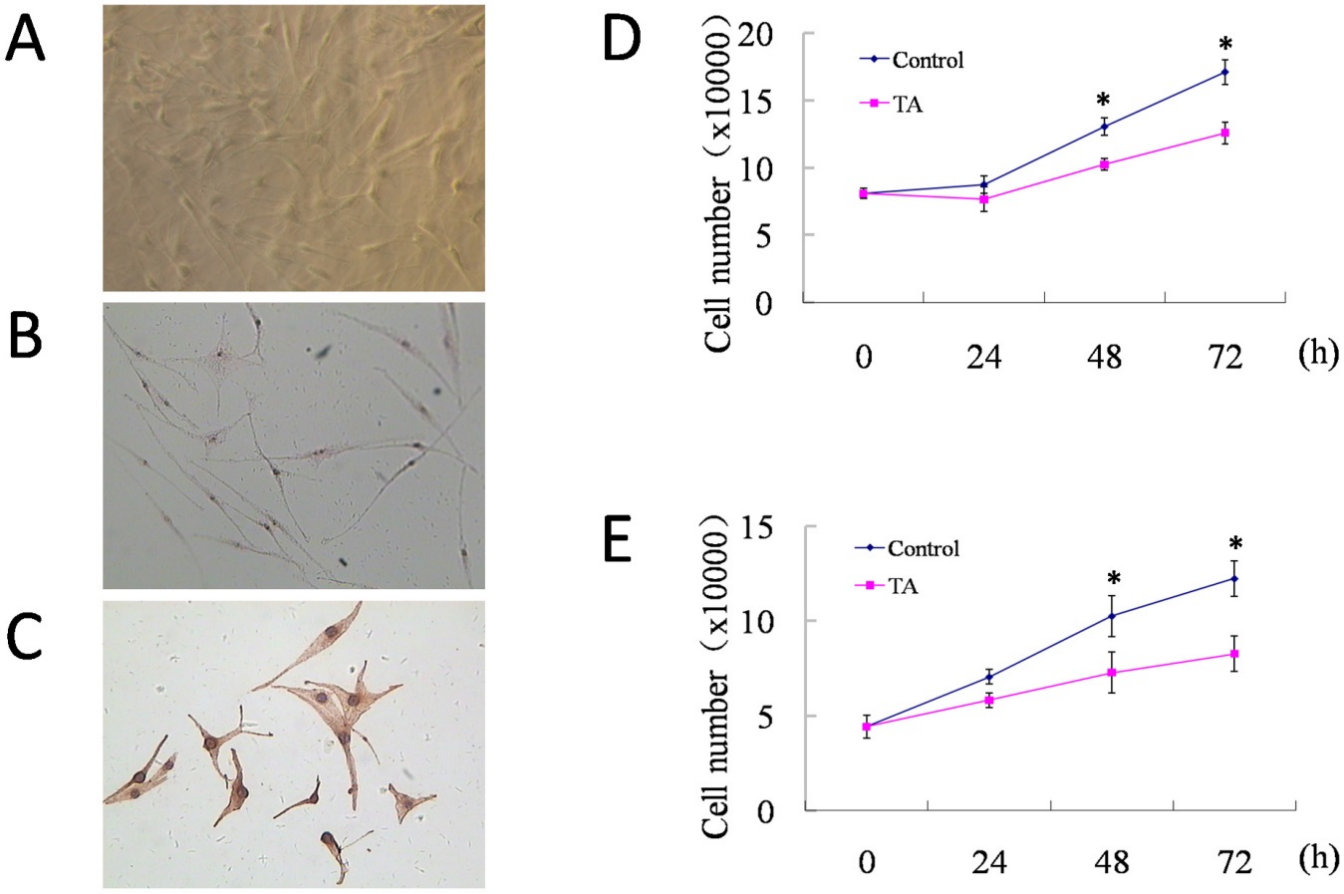
** Normal human melanocytes were cultured and proved to be functionally active, and 1 mg/ml TA inhibited proliferation of both HUVECs and melanocytes without obvious toxicity. (A)** The growth of melanocytes was observed by inverted microscope at 7 days of culture (×40). Melanocytes were identificated by **(B)** DOPA staining (×40) and **(C)** S-100 protein immunohistochemical staining (DAB, ×200). **(D)** Cell counting of HUVECs at 0 h, 24 h, 48 h and 72 h of culture respectively, with or without incubation of 1 mg/ml TA. **(E)** Cell counting of melanocytes at 0 h, 24 h, 48 h and 72 h of culture respectively, with or without incubation of 1 mg/ml TA. TA, tranexamic acid. * *P* < 0.05, Control* vs*. TA group at the same time points. Data are presented as the mean ± SD of three independent experiments.

Both HUVECs and normal human melanocytes were successfully cultured. The melanocytes were multi-dendritic, without contamination of epidermal keratinocytes or dermal fibroblasts (Fig. 1A). Dopa staining (Fig. 1B) and immunohistochemical staining for S-100 protein (Fig. 1C) were positive, proving them to be functionally active. Then, cell counting of HUVECs and melanocytes was performed at 0 h, 24 h, 48 h and 72 h of culture respectively, with or without incubation of 1 mg/ml TA. As shown in Fig. D and E, compared with their control groups, both HUVECs and melanocytes that were treated by 1 mg/ml TA showed similar but slower growth curves, and after 48-72 h of culture, TA significantly inhibited cell growth of both HUVECs and melanocytes without obvious cellular toxicity.

### TA inhibited VEGF165-induced over-expression and activation of VEGF receptors in HUVECs

**Figure 2:**
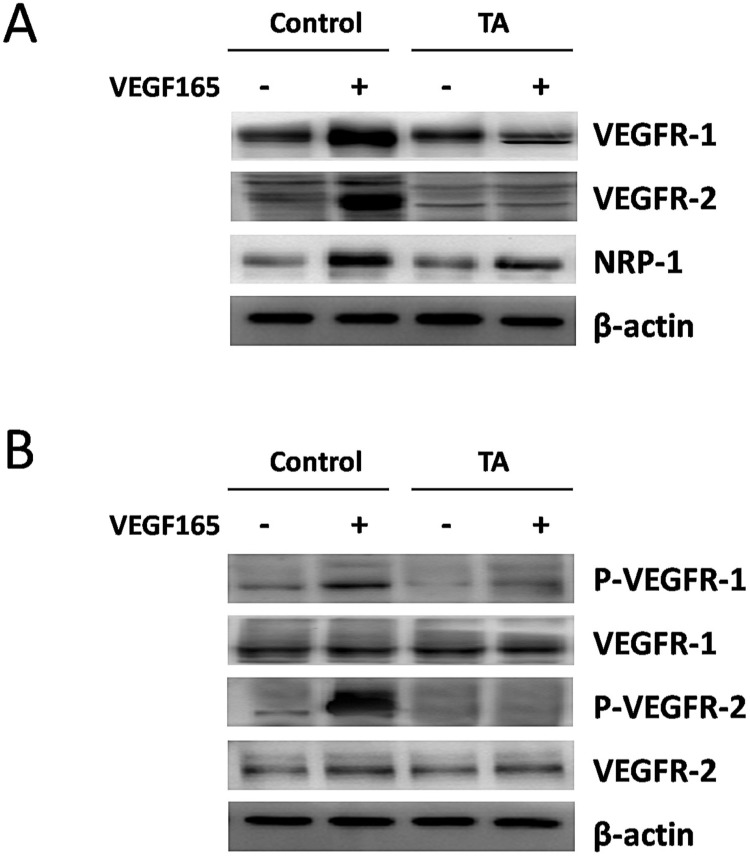
** TA inhibited VEGF165-induced over-expression and activation of VEGF receptors in HUVECs. (A)** The expression of VEGFR-1, VEGFR-2 and NRP-1 in HUVECs in response to 0 or 10 ng/ml VEGF165 with or without incubation of 1 mg/ml TA for 48 h. **(B)** The tyrosine phosphorylation of VEGFR-1 and VEGFR-2 in HUVECs in response to 0 or 10 ng/ml VEGF165 for 5 min with or without pre-incubation of 1 mg/ml TA for 48 h. TA, tranexamic acid. P-VEGFR-1, phospho-VEGFR-1. P-VEGFR-2, phospho-VEGFR-2. β-actin was served as loading control for protein normalization.

As VEGF is natural ligand for VEGFRs, of which the most abundant form is VEGF165 24, we then examined the regulation of VEGFR-1, VEGFR-2 and NRP-1 protein expression in HUVECs by VEGF165 with or without incubation of 1 mg/ml TA for 48 h. As shown in Fig. 2A, a significant induction of VEGFR-1, VEGFR-2 and NRP-1 expression was detected 48 h after 10 ng/ml VEGF165 stimulation. However, this effect was attenuated by 1 mg/ml TA, suggesting that TA could inhibit the enhanced expression of all the above VEGFRs induced by VEGF165 in HUVECs.

We next examined if TA could affect the tyrosine phosphorylation of VEGFR-1 and VEGFR-2 induced by VEGF165 in HUVECs. As expected, 10 ng/ml VEGF165 promoted tyrosine phosphorylation of VEGFR-1 and VEGFR-2 in HUVECs 5 min after stimulation, while no similar effect was observed in cells who were pre-incubated with 1 mg/ml TA for 48 h (Fig. 2B), suggesting that TA could abolish the activation of VEGFR-1 and VEGFR-2 induced by VEGF165 in HUVECs.

### TA inhibited cell proliferation, migration, invasion and tube formation of HUVECs

**Figure 3:**
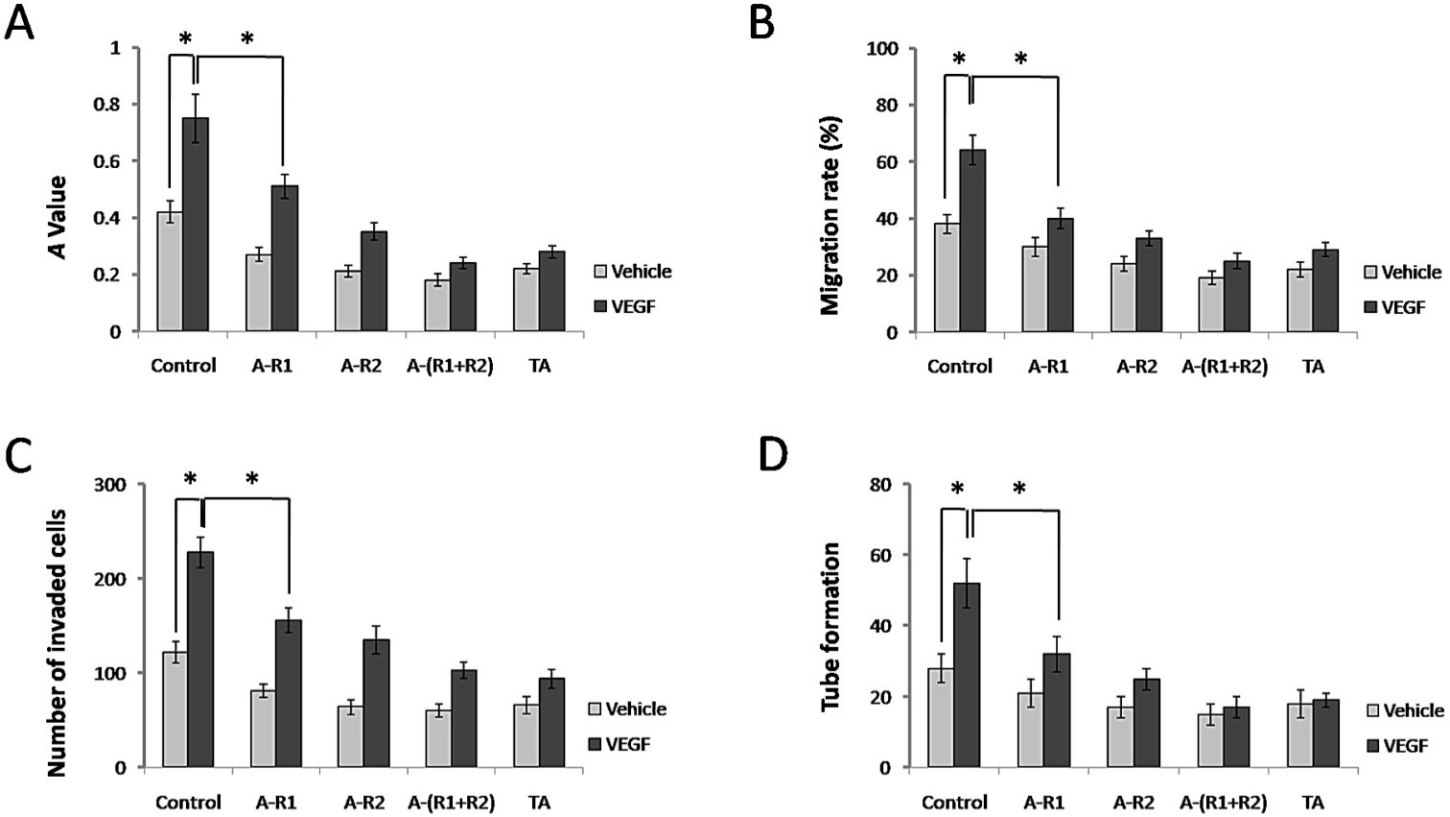
** TA showed similar effects to neutralization of VEGFR-1 and VEGFR-2 in inhibiting cell proliferation, migration, invasion and tube formation of HUVECs. (A)** The *A* value of HUVECs 48 h after 0 or 10 ng/ml VEGF165 stimulation with or without incubation of 1 mg/ml TA, VEGFR-1 and/or VEGFR-2 neutralizing antibody (5 µg/ml, respectively). **(B)** The migration rate of HUVECs 48 h after 0 or 10 ng/ml VEGF165 stimulation with or without incubation of 1 mg/ml TA, VEGFR-1 and/or VEGFR-2 neutralizing antibody (5 μg/ml, respectively). **(C)** The number of invaded HUVECs 48 h after 0 or 10 ng/ml VEGF165 stimulation with or without incubation of 1 mg/ml TA, VEGFR-1 and/or VEGFR-2 neutralizing antibody (5 μg/ml, respectively). **(D)** Tube formation of HUVECs 48 h after 0 or 10 ng/ml VEGF165 stimulation with or without incubation of 1 mg/ml TA, VEGFR-1 and/or VEGFR-2 neutralizing antibody (5 µg/ml, respectively). * *P* < 0.05. TA, tranexamic acid. VEGF, VEGF165. A-R1, VEGFR-1 neutralizing antibody. A-R2, VEGFR-2 neutralizing antibody. A-(R1+R2), VEGFR-1 & VEGFR-2 neutralizing antibodies.

It is well known that VEGF, via VEGFRs in endothelial cells, acts as an essential factor for normal and aberrant angiogenesis 24. We next examined whether TA further interferes with the classical roles of VEGF-VEGFR signaling in blood vessel formation *in vitro*. As shown in Fig. 3A, 10 ng/ml VEGF165 promoted cell proliferation of HUVECs, while neutralization of VEGFR-1 and/or VEGFR-2 significantly decreased this effect. Interestingly, TA showed a similar effect to that by neutralization of VEGFR-1 and VEGFR-2 in HUVECs, as it also significantly decreased cell proliferation of HUVECs induced by VEGF165. VEGF165 also promoted cell migration of HUVECs, and TA exerted a similar effect to that by neutralization of VEGFR-1 and VEGFR-2, because both of them significantly decreased cell migration of HUVECs induced by VEGF165 (Fig. 3B). In addition, VEGF165 accelerated cell invasion of HUVECs, which was inhibited by TA as well as by neutralization of VEGFR-1 and/or VEGFR-2 (Fig. 3C). Tube formation of HUVECs was also greatly facilitated by VEGF165, while TA again exerted a similar effect to that by neutralization of VEGFR-1 and/or VEGFR-2 which significantly attenuated tube formation of HUVECs (Fig. 3D), suggesting that TA could inhibit angiogenesis via targeting VEGFR signaling in HUVECs *in vitro*.

### TA inhibited VEGF165-induced over-expression and activation of VEGF receptors in melanocytes

**Figure 4:**
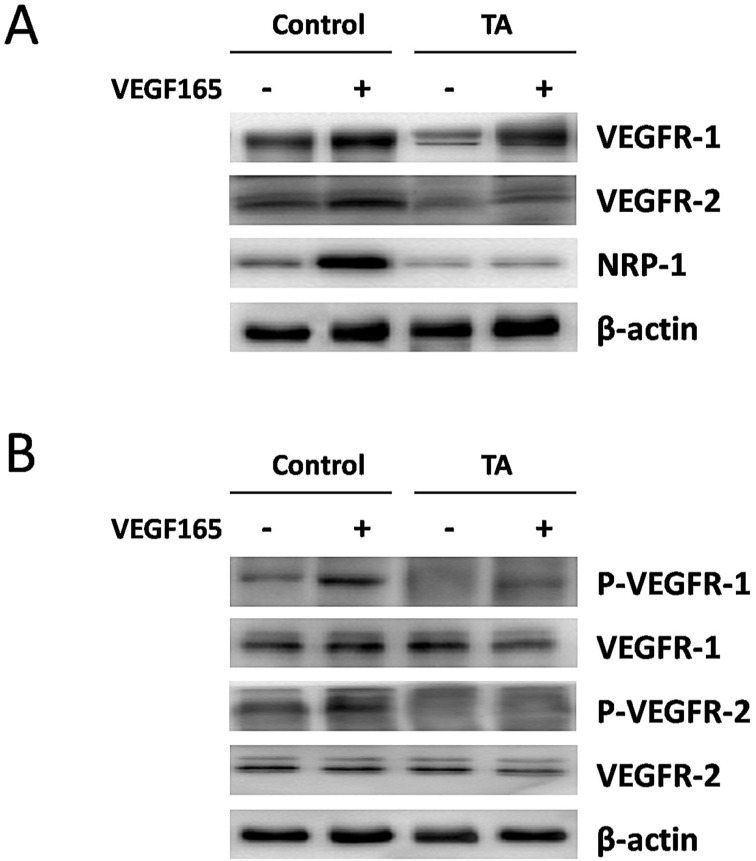
** TA inhibited VEGF165-induced over-expression and activation of VEGF receptors in melanocytes. (A)** The expression of VEGFR-1, VEGFR-2 and NRP-1 in melanocytes in response to 0 or 10 ng/ml VEGF165 with or without incubation of 1 mg/ml TA for 48 h. **(B)** The tyrosine phosphorylation of VEGFR-1 and VEGFR-2 in melanocytes in response to 0 or 10 ng/ml VEGF165 for 5 min with or without pre-incubation of 1 mg/ml TA for 48 h. TA, tranexamic acid. P-VEGFR-1, phospho-VEGFR-1. P-VEGFR-2, phospho-VEGFR-2. β-actin was served as loading control for protein normalization.

We next turned to examine the regulation of VEGFR-1, VEGFR-2 and NRP-1 protein expression by VEGF165 in normal human melanocytes with or without incubation of 1 mg/ml TA for 48 h. As shown in Fig. 4A, an enhanced expression of all the above VEGF receptors was detected 48 h after 10 ng/ml VEGF165 stimulation, but this effect was again attenuated by 1 mg/ml TA, suggesting that TA could also inhibit VEGF165-induced over-expression of VEGFRs in melanocytes.

We then studied if TA could affect VEGF165-induced tyrosine phosphorylation of VEGFR-1 and VEGFR-2 in melanocytes. As shown in Fig. 4B, tyrosine phosphorylation of VEGFR-1 and VEGFR-2 was also promoted 5 min after 10 ng/ml VEGF165 stimulation in melanocytes, while no similar effect occurred in cells that were pre-incubated with 1 mg/ml TA for 48 h, suggesting that TA could also abolish VEGF165-induced activation of VEGFR-1 and VEGFR-2 in melanocytes.

### TA inhibited cell proliferation, tyrosinase activity and melanin production of melanocytes

**Figure 5:**
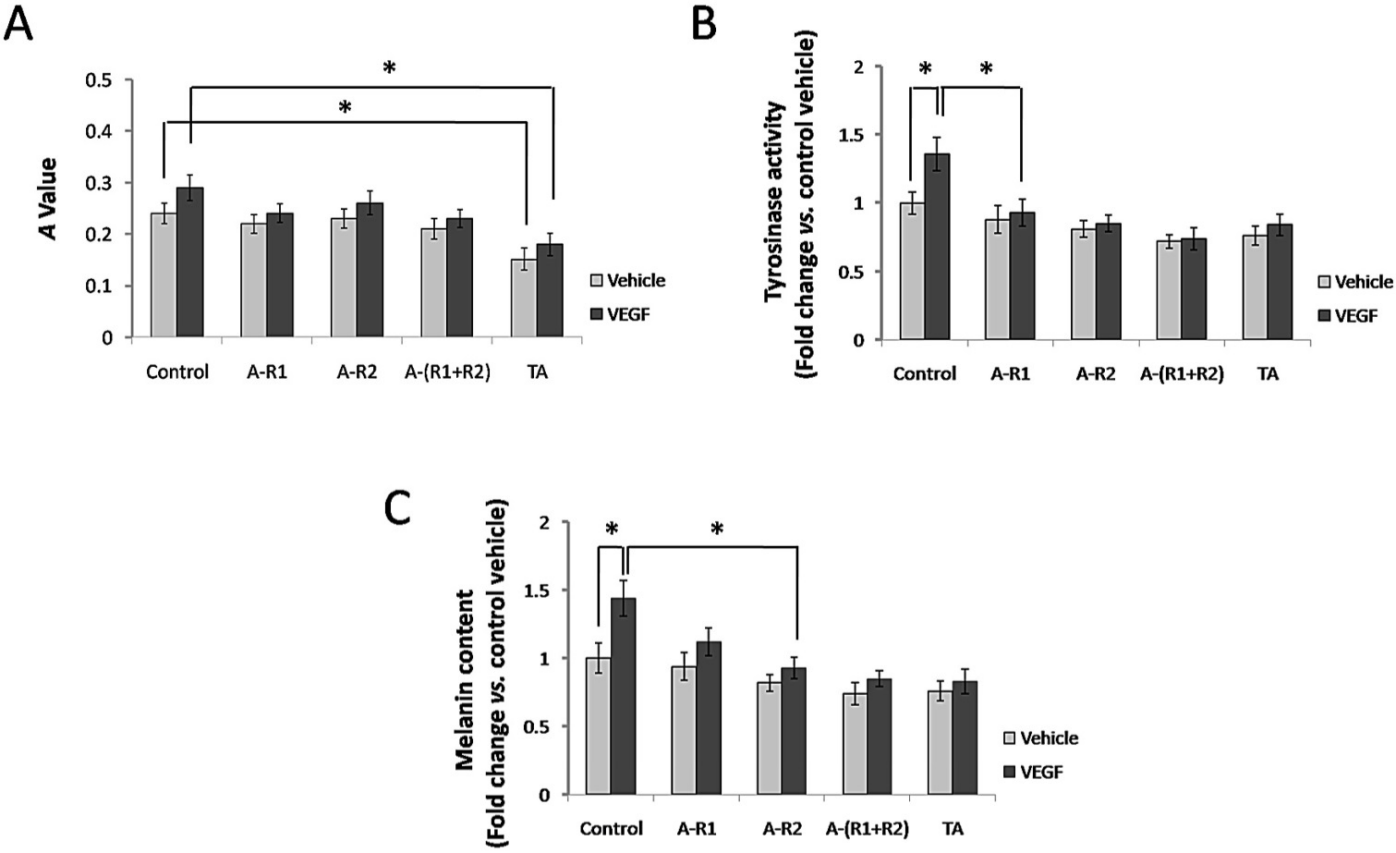
** TA showed similar effects to neutralization of VEGFR-1 and VEGFR-2 in inhibiting tyrosinase activity and melanin production of melanocytes. (A)** The *A* value of melanocytes 48 h after 0 or 10 ng/ml VEGF165 stimulation with or without incubation of 1 mg/ml TA, VEGFR-1 and/or VEGFR-2 neutralizing antibody (5 μg/ml, respectively). **(B)** The tyrosinase activity of melanocytes 48 h after 0 or 10 ng/ml VEGF165 stimulation with or without incubation of 1 mg/ml TA, VEGFR-1 and/or VEGFR-2 neutralizing antibody (5 μg/ml, respectively). **(C)** The melanin production of melanocytes 48 h after 0 or 10 ng/ml VEGF165 stimulation with or without incubation of 1 mg/ml TA, VEGFR-1 and/or VEGFR-2 neutralizing antibody (5 μg/ml, respectively). * *P* < 0.05. TA, tranexamic acid. VEGF, VEGF165. A-R1, VEGFR-1 neutralizing antibody. A-R2, VEGFR-2 neutralizing antibody. A-(R1+R2), VEGFR-1 & VEGFR-2 neutralizing antibodies.

Situated at the basal layer of the epidermis, melanocytes are responsible for melanin synthesis and skin color. Thus, we investigated if TA further interferes with cell proliferation, tyrosinase activity and melanin synthesis of melanocytes via VEGF-VEGFR signaling* in vitro*. As shown in Fig. 5A, 10 ng/ml VEGF165 failed to significantly promote cell proliferation of melanocytes, and neutralization of VEGFR-1 and/or VEGFR-2 showed non-effective for melanocyte proliferation induced by VEGF165. However, TA could still significantly decrease cell proliferation of melanocytes independent of VEGFRs. VEGF165 promoted tyrosinase activity of melanocytes, and TA exerted a similar effect to that by neutralization of VEGFR-1 and VEGFR-2, as both of them significantly decreased tyrosinase activity of melanocytes induced by VEGF165 (Fig. 5B). In addition, VEGF165 enhanced melanin production of melanocytes, which was reduced by TA as well as by neutralization of VEGFR-1 and VEGFR-2 (Fig.5C), suggesting that TA could inhibit melanogenesis via targeting VEGFR signaling in melanocytes *in vitro*.

### TA inhibited the expression of melanogenic proteins in melanocytes

**Figure 6:**
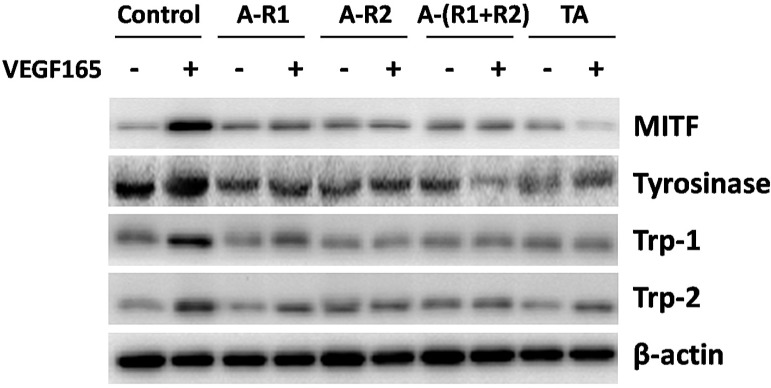
** TA showed similar effects to neutralization of VEGFR-1 and VEGFR-2 in inhibiting the expression of melanogenic proteins in melanocytes.** Western blotting detection of MITF, tyrosinase, Trp-1 and Trp-2 in melanocytes 48 h after 10 ng/ml VEGF165 stimulation with or without incubation of 1 mg/ml TA, VEGFR-1 and/or VEGFR-2 neutralizing antibody (5 μg/ml, respectively). TA, tranexamic acid. A-R1, VEGFR-1 neutralizing antibody. A-R2, VEGFR-2 neutralizing antibody. A-(R1+R2), VEGFR-1 & VEGFR-2 neutralizing antibodies. β-actin was served as loading control for protein normalization.

Melanocytes are responsible for melanin synthesis, which is a complex process requiring melanogenic factors including microphthalmia- associated transcription factor (MITF), tyrosinase, tyrosinase related protein-1 (Trp-1), and Trp-2 25. To elucidate if they are downstream proteins of VEGF receptors, melanocytes were stimulated with 10 ng/ml VEGF165 in the absence or presence of the VEGFR-1 and/or VEGFR-2 neutralizing antibody. As shown in Fig. 6, VEGF165 induced a strong over-expression of MITF, tyrosinase, Trp-1, and Trp-2 in melanocytes, which could be reduced by neutralization of VEGFR-1 and/or VEGFR-2. TA also significantly decreased the over-expression of MITF, tyrosinase, Trp-1 and Trp-2 induced by VEGF165. This suggested that over-expression of melanogenic proteins induced by VEGF165 involved activation of VEGFR-1 and VEGFR-2, and TA could decrease tyrosinase activity and melanin production via inhibiting activation of VEGF receptors and subsequent expression of melanogenic proteins in melanocytes.

## Discussion

Melasma is a common but complex skin condition. Histologically, melasma can display increased epidermal and/or dermal pigmentation, enlarged melanocytes, increased melanosomes, and an increase in dermal blood vessels 1, 2, 21. Multiple etiologies including UV exposure and family history have been implicated in the pathogenesis of this disorder 21. VEGF, an angiogenic factor released from keratinocytes after UV damage, can sustain human melanocytes in tissue culture, which is proposed as one of the mechanisms for the increased activity of melanocytes in melasma 23. Family history is another important risk factor for melasma, no genome-wide study has been performed to examine associated genes, but current findings would suggest that the genes responsible involve pigmentary, inflammatory, hormonal, and vascular responses 26. With increased recognition that angiogenesis may play a role in the pathogenesis of melasma, a copper bromide anti-angiogenesis laser was found to significantly decrease the MASI score and expression of endothelin-1 and VEGF on immunohistochemistry 27. The plasmin inhibitor, TA, has been proved to be effective in treatment of melasma with still unclear mechanisms, one study showed that tranexamic acid may also decrease VEGF and entothelin-1, both of which are responsible for increasing vascularity in affected lesions of melasma 28.

Based on the above knowledge, we speculated that a connection between dermal vessels and cutaneous pigmentation could exist in the pathogenesis of melasma through VEGF receptors (VEGFRs) that are expressed both in endothelial cells and melanocytes. Meanwhile, in order to elucidate how TA acts on melasma, we performed our experiments to study if TA could regulate VEGFRs and their possible related cell functions. We found that although 1 mg/ml TA inhibited cell growth of HUVECs and melanocytes compared to control cells, no obvious cellular toxicity was detected as both of the two cells were still in a good proliferation state, suggesting it is a safe concentration for next experiments. We then found that TA could inhibit VEGF165-induced over-expression and activation of VEGF receptors in HUVECs. As VEGF acts as an essential factor for normal and aberrant angiogenesis via VEGFRs in endothelial cells, we also investigated whether TA further interferes with the classical roles of VEGF-VEGFR signaling in blood vessel formation *in vitro*. As expected, cell proliferation, migration, invasion and tube formation of HUVECs, which represent the capacity to form blood vessels, were all significantly decreased by VEGFR-1 and/or VEGFR-2 neutralizing antibodies. Interestingly, TA exerted a similar effect to that by neutralization of VEGFR-1 and VEGFR-2 in blood vessel formation of HUVECs, suggesting that TA could inhibit angiogenesis via targeting VEGFRs in HUVECs *in vitro*.

We next turned to examine the regulation of VEGFRs by VEGF165 and TA in normal human melanocytes. TA also inhibited VEGF165-induced over-expression and activation of VEGF receptors in melanocytes, a result similar to that in HUVECs. As melanocytes are responsible for melanin synthesis and distribution within the epidermis, which may involve cell proliferation, tyrosinase activity and melanin production, we investigated if TA could further interfere with these activities of melanocytes via VEGFRs* in vitro*. TA significantly decreased cell proliferation of melanocytes independent of VEGFRs, because neither VEGF165 promoted cell proliferation, nor neutralization of VEGFR-1 and/or VEGFR-2 showed any effect to melanocyte proliferation. VEGF165 promoted tyrosinase activity and melanin production of melanocytes, which was significantly decreased by VEGFR-1 and VEGFR-2 neutralizing antibodies. TA exerted a similar effect to that by neutralization of VEGFR-1 and VEGFR-2, suggesting that TA could also inhibit melanogenesis via targeting VEGFRs in melanocytes *in vitro*. As melanin synthesis is a complex process requiring melanogenic proteins including MITF, tyrosinase, Trp-1 and Trp-2, we also asked if they are downstream factors of VEGFRs. VEGF165 induced a strong over-expression of these proteins, which could be reduced by neutralization of VEGFR-1 and/or VEGFR-2. TA again exerted a similar effect to that by neutralization of VEGFR-1 and VEGFR-2, suggesting that over-expression of melanogenic proteins induced by VEGF165 involved activation of VEGFR-1 and VEGFR-2, and TA could decrease tyrosinase activity and melanin production via inhibiting activation of VEGFRs and subsequent expression of melanogenic proteins in melanocytes.

Melasma is a complex condition involving multiple etiologies, and no animal model is available nowadays for our further *in vivo* study, and almost all our patients in the clinic declined biopsy of melasma lesions for experimental use, so we had only to conduct cell studies and present our *in vitro* data in this report. Our findings indicate that VEGFRs are involved in the angiogenesis and melanogenesis in response to VEGF. VEGFRs might be one of the melanogenic factors, and VEGFRs-related melanocyte hyperfunction might lead to development of melasma. TA has been extensively used for clinical treatment of melasma and other hyperpigmentary skin conditions for over 30 years without severe side effects 28, 29, this may be due to the low treatment dosage compared to that for hemostatic purpose, which might be sufficient to relieve activation of VEGFRs in the lesional skin. Although it is well known that TA inhibits plasmin and TA could reduce melanocyte tyrosinase activity by suppressing the production of prostaglandins and UV-induced melanogens through the suppression of the UV-induced increase in epidermal plasmin activity 3, there was hardly any plasmin or plasminogen detected in the supernatants throughout our* in vitro* cell studies (data not shown), implicating that TA did not act on VEGFRs indirectly through plasmin *in vitro*. Other previous studies showed that TA could suppress UVB-induced melanocyte activation by decreasing the levels of prohormone convertase-2 and α-MSH 30, and TA inhibited melanogenesis by activating the autophagy system in cultured melanoma cells 31, suggesting that TA can affect melanocyte function in other multiple pathways without traditional inhibition of plasmin. In addition, TA did not seem to act through binding with VEGF, as TA alone could inhibit the expression and phosphorylation of VEGFRs without VEGF stimulation (Fig. 2 and 4), and each experiment group for phosphorylation of VEGFRs was performed by pretreatment of TA before VEGF stimulation, there was no chance for TA binding directly with VEGF. So, we imagined that TA might act directly through binding with VEGFRs or indirectly through other molecular signaling. We tried to seek several possible molecular mechanisms or signaling pathways concerning how TA directly interacted with VEGFRs, but we failed to find any positive result. In our opinion, TA largely interacts with VEGFRs indirectly through other undefined molecular signaling, and importantly of course, further work will be needed to make a more definite conclusion. Anyway, our findings can offer new understanding of the pathogenesis of melasma, which may help in the development of future treatments for this common, yet challenging condition.
